# P-1988. Prediction of Clinically-Significant Infections among Stem Cell Transplant Patients Using Next-Generation Sequencing Surveillance

**DOI:** 10.1093/ofid/ofaf695.2155

**Published:** 2026-01-11

**Authors:** Cyrus Ghaznavi, Lakshin Kumar, Emily Lydon, Chaz Langelier, Peter V Chin-Hong, Monica Fung

**Affiliations:** UCSF, San Francisco, CA; University of California San Francisco, San Francisco, California; University of California San Francisco, San Francisco, California; University of California San Francisco, San Francisco, California; UCSF, San Francisco, CA; University of California San Francisco, San Francisco, California

## Abstract

**Background:**

Despite increasing literature on the performance of plasma metagenomic next generation sequencing (pmNGS) for diagnosis of infection among immunocompromised patients, there is scant data regarding the ability of pmNGS to predict infections prior to onset.

**Figure:**
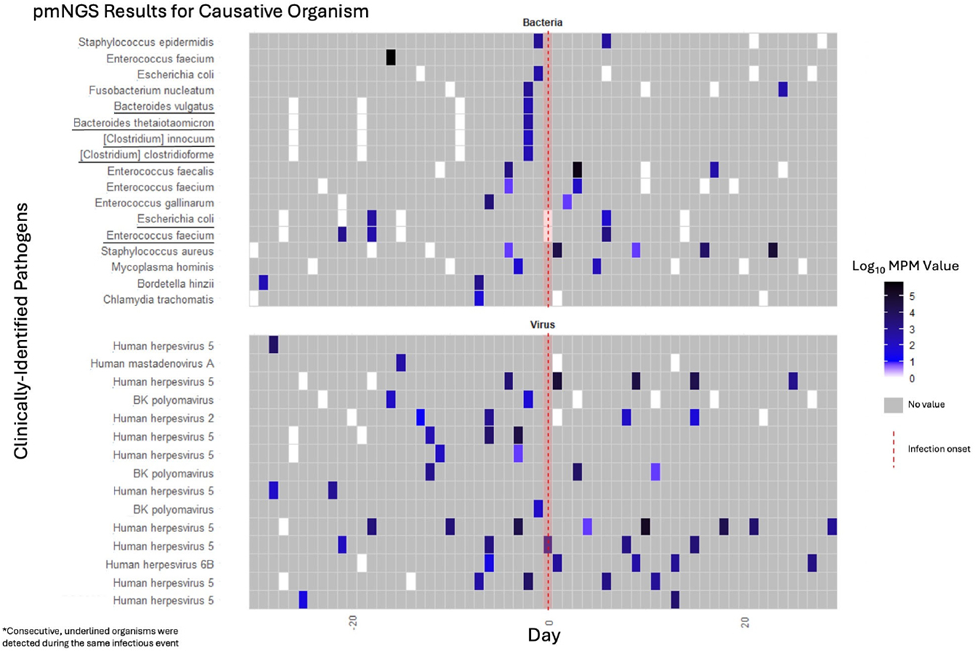
Plasma metagenomic next generation sequencing (pmNGS) detections of causative organisms before and after infection onset Rows on the y-axis correspond to clinically-identified pathogens responsible for infectious episodes that occurred during the DISCOVER trial. Each colored cell corresponds to the Log10(molecules per microliter) for that particular organism based on pmNGS (Karius) testing. The x-axis displays the relative time before and after infection onset, delineated by the dashed red line, in days. Grey cells correspond to dates during which no pmNGS testing was performed; white cells correspond to dates in which pmNGS testing was performed but the causative organism was not detected.

**Methods:**

We retrospectively analyzed data from the DISCOVER trial (NCT02804464), which enrolled 70 allogeneic stem cell transplant (SCT) patients who underwent serial pmNGS (Karius) testing post-SCT for 1 year (weekly surveillance for 7 weeks then monthly; for diagnosis within 24h of fever). Infectious events and onset were identified by retrospective chart review and ID specialist adjudication of standard of care testing including standard microbiologic testing (SMT) and pmNGS.

**Results:**

During the study period, there were 28 (15 viral and 13 bacterial) unique clinical episodes of infection with positive mNGS testing prior to infection onset (21 SMT+/pmNGS+, 7 SMT-/pmNGS+). Among the 15 viral and 13 bacterial infections, SMT also identified the infectious etiology in 13 and 8 cases, respectively. Among the 15 pmNGS+ viral events, the most recent pmNGS detected the clinically-identified viral pathogen an average of 9.2 days before infection onset (range: 1 to 28 ). Among the 13 pmNGS+ bacterial infections, the most recent pmNGS detected the adjudicated bacterial pathogen an average of 5.7 days in advance (range: 1 to 18 ). Among the 15 pmNGS+ viral events, the most recent new pmNGS detection (the first detection after a period of no detection) of the clinically-identified viral pathogen occurred an average of 17.4 days before infection onset (range: 1 to 56). Among the 13 pmNGS+ bacterial events, the most recent new pmNGS detection of the adjudicated bacterial pathogen occurred an average of 12.0 days before infection onset (range: 1 to 57). In general, viral organisms were detected earlier than bacterial organisms.

**Conclusion:**

Plasma mNGS testing can detect pathogens prior to clinically-significant infectious events among SCT patients. Further studies are needed to understand the clinical utility of pmNGS for predicting infections.

**Disclosures:**

Monica Fung, MD, MPH, Karius, Inc: Advisor/Consultant|Scynexis, Inc: Grant/Research Support

